# Improving Cry8Ka toxin activity towards the cotton boll weevil (*Anthonomus grandis*)

**DOI:** 10.1186/1472-6750-11-85

**Published:** 2011-09-09

**Authors:** Gustavo R Oliveira, Maria CM Silva, Wagner A Lucena, Erich YT Nakasu, Alexandre AP Firmino, Magda A Beneventi, Djair SL Souza, José E Gomes, José DA de Souza, Daniel J Rigden, Hudson B Ramos, Carlos R Soccol, Maria F Grossi-de-Sa

**Affiliations:** 1 Departamento de Biologia Celular, Universidade de Brasília - UnB, Brasília, DF, Brasil; 2 Embrapa Algodão - Campina Grande, PB, Brasil; 3 Embrapa Recursos Genéticos e Biotecnologia, PqEB- Final W5 Norte -Brasília, DF, Brasil; 4 Institute of Integrative Biology, University of Liverpool, Liverpool, UK; 5 Programa de Pós-graduação em Biologia Celular e Molecular, UFRGS, Porto Alegre, RS, Brasil; 6 Pós-Graduação em Ciências Genômicas e Biotecnologia - UCB, Brasília, DF, Brasil; 7 Programa de Pós-Graduação em Processos Biotecnológicos-UFPR, Curitiba, PR, Brasil

**Keywords:** *Anthonomus grandis*, *Bacillus thuringiensis*, Cotton, DNA shuffling, Phage display, Molecular modeling

## Abstract

**Background:**

The cotton boll weevil (*Anthonomus grandis*) is a serious insect-pest in the Americas, particularly in Brazil. The use of chemical or biological insect control is not effective against the cotton boll weevil because of its endophytic life style. Therefore, the use of biotechnological tools to produce insect-resistant transgenic plants represents an important strategy to reduce the damage to cotton plants caused by the boll weevil. The present study focuses on the identification of novel molecules that show improved toxicity against the cotton boll weevil. *In vitro *directed molecular evolution through DNA shuffling and phage display screening was applied to enhance the insecticidal activity of variants of the Cry8Ka1 protein of *Bacillus thuringiensis*.

**Results:**

Bioassays carried out with *A. grandis *larvae revealed that the LC_50 _of the screened mutant Cry8Ka5 toxin was 3.15-fold higher than the wild-type Cry8Ka1 toxin. Homology modelling of Cry8Ka1 and the Cry8Ka5 mutant suggested that both proteins retained the typical three-domain Cry family structure. The mutated residues were located mostly in loops and appeared unlikely to interfere with molecular stability.

**Conclusions:**

The improved toxicity of the Cry8Ka5 mutant obtained in this study will allow the generation of a transgenic cotton event with improved potential to control *A. grandis*.

## Background

*Anthonomus grandis *(Coleoptera: Curculionidae), commonly known as the cotton boll weevil, is the most harmful cotton-feeding insect-pest in the Americas [1-4]. Even in cotton fields where the insect has been considered eradicated (e.g. Mississipi State USA), the growers must maintain a continued vigil because of the tremendous ability of the boll weevil to return to previous controlled regions from time to time to re-infest. Millions of dollars in insecticides were spent by growers to have areas eradicated. In not eradicated areas, yield losses to boll weevil exceeded until 10 percent in certain years and the growers had to apply 8-12 boll weevil sprays per field [5]. These repeated treatments are necessary, because only adult boll weevils feed on tender cotton terminals, on pollen from flower buds and on fruits. The larvae are protected inside the boll, which makes difficult the contact with chemical pesticides. Recently presented during forum Brazil' 2010/11, cotton area production is estimated at 1.2 million of hectares the highest since the early 1990's [6]. However, especially in Brazil, boll weevil is considered a key pest in cotton fields and despite of pest management and several insecticide sprays, an efficient boll weevil control does not exist. As an alternative to chemicals, the use of transgenic plants resistant to *A. grandis *presents a potential solution to the problem posed by insect-pests [7]. The development of genetically modified (GM) crops expressing Cry toxins has been widely researched due to the toxic effect of these molecules against insect-pests (lepidopterans, coleopterans and dipterans) and nematodes which attack and thereby affect the productivity of crops such as cotton [8]. These toxins are harmless to vertebrates and plants due their high specificity to the insect target. Since 1996, several insect-resistant GM cotton events have been used commercially throughout the world, including Bollgard^® ^(expressing the *cry1Ac *protein) and Bollgard II^® ^(expressing the *cry1Ac + cry2Ab *proteins) from Monsanto, Widestrike^® ^(expressing the *cry1Ac + cry1F *proteins) from Dow Agrosciences and VipCot^® ^(expressing *Vip3A *protein) from Syngenta/Deltapines. This technology has been used to control lepidopterans, allowing an increase in productivity, reductions in production costs, human intoxication and environmental damage due to a reduction in chemical pesticide application [9]. At the moment, however, none of the commercially available GM cotton events are effective against the cotton boll weevil, which is the most harmful cotton insect-pest in Latin America. Cry proteins are included in parasporal crystalline structures produced by *Bacillus thuringiensis *during sporulation [10,11]. Several hypothesis about the insecticidal mechanism of action of Cry toxins have been discussed in previous studies [12,13], including the pore formation model [8,13,14] and the signal transduction model [15]. More recently, Guo et al [16] proposed a plausible model for the initiation of Cry toxin domain disassembly before membrane penetration and pore formation. After ingestion, the crystals are solubilised and activated by insect midgut proteases. According to Bravo and Soberon [8], the binding of toxins to specific cadherin-like receptors triggers the oligomerization of toxin monomers. Directed by an aminopeptidase-N receptor, the oligomer is inserted into the cell membrane and forms a pore. Those pores are responsible for a net influx of ions and water, leading to disturbances in osmotic balance, cell lysis, midgut damage and insect death [8]. In the intracellular signalling model [15], the binding of toxins to the cadherin-like receptor triggers an Mg^2+^-dependent cAMP signalling pathway that promotes cell death. In both models, the affinity for cadherin receptors has been reported as the main step that determines specificity. Mutations in those receptors have been reported to be the cause of resistance acquisition [8]. Moreover, Broderick et al [17] suggested that the cry toxin activity is also dependent on the microorganisms of the insect midgut. Molecular strategies that involve structural and biochemical studies of Cry toxins, as well as the isolation and characterisation of new *cry *genes, are necessary to help elucidate the mechanisms of action of Cry toxins and also to select molecules that have the potential for improved toxicity and specificity [14].

In this context, DNA shuffling, as described by Stemmer [18] and following modifications reported by Zhao and Arnold [19], is the most commonly used technique to promote *in vitro *directed molecular evolution. In this technique, the introduction of random mutations into the nucleotide sequences results in a pool of mutants from which proteins with improved function can be selected. Considering the biotechnological context, this technique has been used as a tool to generate libraries that contain variant genes encoding engineered proteins, which can subsequently be selected according to their potential agricultural or pharmaceutical use [20,21]. In a previous study reported by our group, a recombinant toxin produced by the *cry1Ia12 *gene exhibited moderate toxicity towards first instar larvae from both the lepidopteran fall armyworm (*Spodoptera frugiperda*) (50% mortality with 5 μg.mL^-1^) and the coleopteran cotton boll weevil (50% mortality with 230 μg. mL^-1^) [22]. More recently, *cry1Ia12synth *(a *cry1Ia12 *synthetic gene containing plant codon usage) was used in an approach applying DNA shuffling coupled with the phage display technique, which involves the presentation of peptide and protein libraries on the surface of phage particles for the facilitated selection of proteins with high affinity and specificity for a determined target [23]. Our data showed that this strategy was able to efficiently generate genetic diversity, and the screening of the combinatorial library for *cry1Ia12synth *variants resulted in the identification of novel molecules with improved entomotoxicity towards the sugarcane giant borer larvae, *Telchin licus licus *(Lepidoptera: Castniidae), an activity not exhibited by the wild-type protein encoded by the original *cry1Ia12 *gene [24].

In a parallel manner, our team isolated a new gene classified as *cry8Ka1 *(GenBank accession no. FJ422558) from a Bt strain that showed moderate toxicity to the cotton boll weevil [25]. The entomotoxicity of recombinant Cry8Ka1 protein expressed in *E. coli *bioassayed against *A. grandis *larvae was confirmed [25,4].

In the present study, the *cry8Ka1 *gene was used in the combined DNA shuffling and phage display techniques to create novel Cry mutant toxins with improved activity towards the coleopteran *A. grandis*. Around 10^5 ^Cry8Ka1 variants were generated, and the combinatorial library and screened phage selection allowed the isolation of new Cry toxins that exhibit improved toxicity against the boll weevil insect-pest. Among the screened Cry variants, Cry8Ka5 was selected for its high toxicity and potential use as a biotechnology tool for the generation of transgenic cotton plants. Analyses from theoretical models created for Cry8Ka1 and Cry8Ka5 toxin and comparison with other Cry toxin structures revealed that essential structural features are conserved.

## 2. Methods

### 2.1 Preparation of A. grandis Brush Border Membrane Vesicles (BBMVs)

Neonate *A. grandis *larvae were obtained from colonies grown at the Embrapa Genetic Resources and Biotechnology Centre (Brasilia, Brazil) using an artificial diet [25]. The BBMVs were prepared from midguts of boll weevil larvae by Mg/EGTA precipitation and differential centrifugation [26]. The larval midguts were extracted and transferred to a microcentrifuge tube with MET (300 mM mannitol, 5 mM EGTA, 17 mM Tris, pH 7.5) containing 1 mM PMSF. Two hundred midguts were centrifuged at 2500 × *g *for 5 min at 4°C, and the pellet was washed twice with MET buffer. The concentration of BBMVs proteins was determined by the Bradford assay [27] using bovine serum albumin (BSA) as the standard for the calibration curve. The aliquots were stored to -80°C until use.

### 2.2 cry8Ka1 gene amplification

A *cry8Ka1 *gene was previously isolated by our team from a collection of *Bacillus thuringiensis *at the Embrapa Genetic Resources and Biotechnology [25] was chosen for further studies because the encoded toxin had the potential to control the cotton boll weevil (*A. grandis*). First, the *cry8Ka1 *gene (2001 bp), corresponding only to the active part of the toxin, was PCR-amplified from the original plasmid using Platinum *Taq *DNA Polymerase High Fidelity (Invitrogen) and the Cry8Ka1sfiF (5'CCCGGCCCAGGCGGCCGACCACGCGTATCGA 3') and Cry8Ka1sfiR (5'CCCGGCCGGCCTGGCCGTTCAAGGAACCGTT 3') primers, which introduced an *Sfi *I restriction site (underlined). The PCR program included the following steps: a denaturation step, consisting of 1 cycle at 95°C for 5 min; 29 cycles consisting of a denaturation step of 40 s at 95°C; a hybridisation step of 40 s at 45°C; an elongation step of 40 s at 72°C; and finally, a step consisting of 2 min at 72°C. The PCR amplification product was analysed by 1% agarose (w/v) gel electrophoresis, and the DNA (approximately 2000 bp) was excised and gel-purified using the Geneclean^® ^II Kit (BIO 101).

### 2.3 Generation of a combinatorial library using DNA shuffling and phage display

The *Sfi *I-digested, PCR-amplified *cry8Ka1 *gene was used as the starting material for the DNA-shuffling procedure [18,19]. First, 10 μg of the purified *cry8Ka1 *gene was randomly digested in a mixture containing 70 U of DNAse I enzyme (Invitrogen) in DNase I buffer (50 mM Tris buffer, pH 7.6, containing 1 mM MnCl2 and 0.1 mg/mL BSA). The digestion reaction was performed at 15°C for 20 min and interrupted by the addition of 5 μL of 0.5 M EDTA. The digestion product was analysed by 2.5% agarose (w/v) gel electrophoresis and the 30-50-bp fragments were jointly purified using the High Pure PCR Product Purification Kit (ROCHE). Ten microliters of purified product were obtained by performing a PCR without primers in a 25-μL final volume containing 2.5 μM of each dNTP, 0.5 mM MgSO_4_, and 2.5 U of Platinum^® ^*Taq *DNA Polymerase High Fidelity (Invitrogen) in the supplied 1X buffer. A PCR program consisting of the following steps was used: 95°C for 2 min; 43 cycles of 95°C for 1 min; 44°C for 1 min and 72°C for 1 min (with a 5-s increase in extension time per cycle); and 7 min at 72°C. To reassemble the variant genes, the product of the primer-less PCR (7.6 μL) was used as template for a second PCR using the Cry8Ka1SfiF and Cry8Ka1SfiR primers. The PCR mixture had a final volume of 500 μL and contained 0.2 mM of each dNTP, 2 mM MgSO_4_, 800 nM of each primer and 25 U of *Taq *(Phoneutria)/Platinum^® ^*Taq *DNA Polymerase High Fidelity (Invitrogen) (1:1 mixture) in the supplied 1X Platinum Taq buffer. The conditions for the second PCR were as follows: 2 min at 95°C; 10 cycles of 30 s at 95°C; 30 s at 45°C and 1 min at 72°C; 14 cycles of 30 s at 95°C; 30 s at 43°C and 42 s at 72°C (with a 20-s increase per cycle); and finally, 7 min at 72°C. All assembly reactions were performed in a Mastercycler gradient thermocycler (Eppendorf). The amplified shuffled product of approximately 2000 bp was analysed on a 1% agarose (w/v) gel, the single band of approximately 2000 bp was excised and the DNA was gel-purified using the Geneclean^® ^II Kit (BIO 101). This product was digested using the *Sfi *I enzyme, and the variant genes (0.9 μg) were ligated into the pComb3X phagemid (3.6 μg) [28], which was also linearised using the *Sfi *I site. For a single reaction, T4 DNA ligase (Invitrogen) (12 U) and 5X ligase buffer were mixed to obtain a 200μL final volume. The ligation product was dialysed, lyophilised and dissolved in 15 μL of water and subsequently fractionated into five aliquots. Each aliquot (3 μL) was used to transform 60 μL of the electrocompetent XL1-Blue^® ^strain of *Escherichia coli *(Stratagene), and the following procedures were carried out as first described by Barbas III et al. [29] using the same modifications that were previously reported in [24].

### 2.4 Selection of the Cry8Ka1 toxin variants that bind to A. grandis BBMVs (biopanning)

The biopanning procedure for screening the combinatorial library and selecting specific *cry8Ka1 *toxin variants fused to phage particles was performed as described by Barbas III et al. [29] with modifications recently reported by Craveiro et al. [24]. At each round, wells in microtitre plate were coated with BBMV preparation (100 μg) and incubated 16 h at 4°C. After five rounds of selection, the clones from the cycle that exhibited the highest number of colony-forming units (cfus) were isolated and analysed to verify the integrity of the inserted *cry8ka1 *variant genes via colony PCR using the Cry8Ka1sfiF and Cry8Ka1sfiR primers (described above). The clones that presented amplicons around 2000 bp in length were further used for the expression of Cry8Ka1 variant proteins.

### 2.5 Expression of Cry8Ka (parental and variant gene toxins) in fusion phage particles

First, the parental *cry8Ka1 *gene and several variants exhibiting size integrity (approximately 2000 bp) were expressed on the surface of M13 phage and analysed by immunodetection (Dot blot) exactly as reported in a previous study using the *cry1Ia12 *gene and variants by Craveiro et al. [24].

### 2.6 Subcloning of cry8Ka1 and selected variant genes into vectors for expression in E. coli bacteria

To confirm that toxins expressed by phage fusion exhibited correct folding and activity, *cry8Ka1 *and *cry8Ka5 *variant genes were subcloned into the pET101/D TOPO (Invitrogen) plasmid according to the manufacturer's instructions. The CRY8PETF (5'CACCATGCGACACCTTCTACATCTG 3') and CRY8PETR (5'CTAAGAAGCGTAGTCCGGAAC 3') primers were used to insert recombinant sites into genes. The pET101-based expression constructions, Cry8Ka1PET101 and Cry8Ka5PET01, were used to transform *E. coli *cells of the BL21 (DE3) strain according to the manufacturer's instructions. One colony of the bacteria carrying each construct was cultivated at 37°C in 5 mL of Luria Bertani (LB) medium containing ampicillin (100 μg mL^-1^) (i.e., LB selective medium), under vigorous agitation (200 rpm) until an OD_600nm _of 0.6 was reached. This pre-inoculum suspension was used to inoculate 2 L of fresh LB selective medium, which was agitated for 6 h after induction by the addition of IPTG to a final concentration of 0.5 mM. Before induction, an aliquot of the culture cells was collected and reserved. Typically, cells were harvested by centrifugation (3000 × *g*, 10 min), and the pellet was resuspended using 1 mL of 20 mM Tris- HCl (pH 8.0) buffer. Thereafter, the cell suspension was lysed by sonication and stored at -80°C until use. Final purification of His6X-tagged mature Cry8Ka1 and variant toxins was performed using affinity chromatography on 5 mL batches of Ni-NTA (QIAGEN) solid phase. The column equilibrium and chromatography conditions were performed according to the manufacturer's instructions. The eluted fractions were collected, dialysed against water and then lyophilised.

### 2.7 Western blotting analysis

The purified recombinant proteins were analysed using SDS-PAGE [30]. Typically, a 12% gel was loaded with 5 μg of each expressed protein (Cry8Ka1 and its selected mutants). After electrophoresis, the proteins in the gel were visualised by Coomassie Blue R-250 staining. For Western blotting, the protein samples were transferred onto a HYBOND™-C EXTRA nitrocellulose membrane using a Trans-blot Semi-dry Transfer Cell (BioRad^®^). The solution used to block the membrane consisted of 2% (w/v) bovine serum albumin (BSA) in TBS (5 mM Tris-HCl, 15 mM NaCl, pH 7.5). Thereafter, the membrane was incubated with anti-His-AP conjugate (Invitrogen^®^) (1:2000 dilution in TBS) for 2 h at room temperature. After the membrane was washed three times using TBS-T (0.05% (v/v) Tween 20 (Sigma^®^) added to TBS), the immune reactive bands were detected by immersing the blot according to the manufacturer's instructions using an alkaline phosphatase conjugate substrate^® ^kit (BioRad^®^).

### 2.8 Quantification of the expressed toxins (ELISA)

To quantify proteins expressed by *E. coli *(as described in *2.6*), enzyme-linked immunosorbent assays (ELISA) were performed using the polyclonal rabbit anti-Cry8Ka1 antibody, which was previously produced and purified by our team (not shown). The concentration of purified proteins was first determined according to Bradford method [27]. For a standard curve, a serial dilution was performed using purified Cry8Ka1 toxin (4 ng to 0.0019 ng) in wells of a microtiter plate. The wells of the plate were protein immobilised for 24 h at 4°C and then blocked using a solution containing 3% (w/v) BSA in TBS-T buffer for 4 h at room temperature. Following the washes, the polyclonal anti-Cry8Ka1 antibody (diluted 1:10000 in TBS-T containing 1% BSA) was deposited and incubated for 16 h at 4°C. After washes, samples were incubated with anti-rabbit IgG secondary antibody conjugated to peroxidase (Bio-Rad) (diluted 1:1000) for 2 h at room temperature. The TMB Peroxidase EIA Substrate Kit^® ^(BioRad) was used for detection according to the manufacturer's instructions, and the reaction was stopped with 1 N H2SO4. The absorbance was measured at 405 nm using Benchmark Plus (BioRad). The assays were carried out in triplicate.

### 2.9 Bioassays of Cry8Ka1 and variants using A. grandis larvae

To assess the toxicity of Cry8Ka1 and its variants against neonate *A. grandis *larvae the bioassays were performed in two different situations. In the first bioassays for fast screening of variants indicating toxicity, fusion phage-expressed Cry8ka1 and variants were utilized in artificial diet (10^11 ^pfu mL^-1 ^of phage-expressed). Later bioassays were repeated using expressed in bacteria and purified Cry8ka1 and variants (0, 3, 6, 9 and 12 μg/mL) showing significant toxicity in preliminary screening. In both, bioassays were carried out in six-well cell culture plates filled with artificial diet [18% (w/v) Agar, 2.72% (w/v) Brewer's yeast, 4.48% (w/v) Soybean protein, 2.72% (w/v) Wheat germ, 18% (w/v) Pharmamedia^® ^, 0.1% (w/v) Sorbic Acid, 0.9% (w/v) Ascorbic Acid, 2.72% (w/v) Glucose, 0.9% (w/v) Nipagin, 0.05% (w/v) Mineral salts, 0.45% (w/v) vitamin mixture] and phage-expressed (first bioassay) or Cry8Ka1 or variants (detected in dot blot analyses, data not shown)(second bioassay). Twelve larvae were placed in each well and the plate was incubated for seven days at 27°C (±1) with 80% relative humidity and a 14-h photoperiod. An artificial diet containing no additional Cry proteins was used as the negative control. Each treatment was carried out in triplicate and the bioassay was repeated to until six different dates. After seven days, the number of surviving larvae was recorded for each treatment. Statistical analyses were performed to compare the average percent mortalities by ANOVA and Tukey's means comparison analysis using the SigmaStat^® ^software vs. 3.1 (Systat Inc., San Jose, California, 2004). The LC50 (lethal concentration required to kill 50% of insects) obtained from bioassays performed using bacteria-expressed proteins was calculated by Probit analysis [31] using the Polo-Pc software (LeOra Software).

### 2.10 DNA sequence analysis

The nucleotide sequences of the *cry8Ka1 *variant genes encoding toxins active against *A. grandis *larvae (according to bioassay analyses) were determined using a 3130xL Genetic Analyser (Applied Biosystems). To obtain the complete nucleotide sequences, several primers were used. 1) Primers designed to determine the N-terminal and C-terminal sequences were as follows: PCOMBF (5'-GCTTCCGGCTCGTATGTTGTGT-3') and PCOMBR (5'-CGTTTGCCATCTTTTCATAAT-3'). 2) Primers designed to obtain the intermediate sequences were as follows: Cry8INTERF (5'-CATATGCACAAGCTGCGAATT-3'), Cry8INTERR (5'-GCTTCCGGCTCGTATGTTGTGT-3'), Cry8ka5INTERF (5'-AGCGGATTTGGGCAATTCAG-3') and Cry8ka5INTERR (5'-TAACAGCTGGAATTTGAGGA-3'). The sequences were analysed using the BLASTn and BLASTp algorithms [32], which are available on the NCBI homepage http://www.ncbi.nlm.nih.gov/blast/Blast.cgi. In order to assemble the sequenced fragments and to identify the position of the mutated residues, multiple sequence alignment programs were used, including the STADEN PACKAGE (available at http://staden.sourceforge.net/) [33] and CLUSTALW (available at http://www2.ebi.ac.uk/clustalw/) [34].

### 2.11 Molecular analysis and homology modelling

Proteins showing homology to Cry8Ka1 were found in the nr database at the National Center for Biotechnology Information (NCBI) using BLAST with default parameters [32]. The resulting sequence set was aligned using Muscle [35]. Suitable templates for Cry8Ka1 model building were found using a FASTA search of sequences in the Protein Data Bank [36] at the European Bioinformatics Institute http://www.ebi.ac.uk/fasta33.

After manual refinement of the sequence alignment, homology models of Cry8Ka1 (667 amino acid residues) and mutant Cry8Ka5 (649 amino acid residues) were created using MODELLER, Version 9.8 [37]. The Cry8Ea1 [16] (PDB entry 3EB7) toxin structures was used as template for construction of the structural model. In the final alignment, Cry8Ka1 shared 34.7% sequence identity with the template. Default regimes of model refinement by energy minimisation and simulated annealing were employed. Because of the low sequence similarity between target and template, a rigorous iterative modelling protocol was adopted in which 50 models were constructed and analysed. These models were analysed for packing and for stereochemical properties using PROCHECK [38]. Possible misalignments were highlighted by DOPE (Discrete Optimised Protein Energy; a staticatical potential used to assess homology models, running within the MODELLER environment) peaks, and variations in alignment of these regions were examined. When no further improvements could be achieved, the model with the best PROCHECK and DOPE scores was taken as the final model. Diagrammatic representations of the structures were generated using PyMOL 1.3 [39] (available at http://www.pymol.org/).

## 3. Results

### 3.1 DNA shuffling and combinatorial library construction

**Figure 1:**
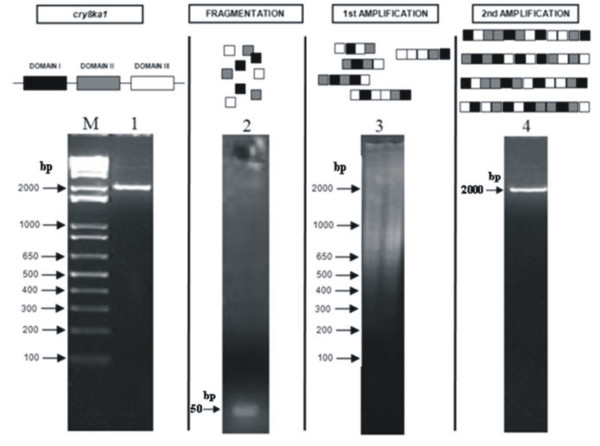
**Illustration of the DNA shuffling steps**. The DNA shuffling product was analyzed on 2.5% agarose gel electrophoresis. **Lane M**: 1Kb-plus ladder (Invitrogen); **lane 1**: *cry8Ka1 *gene amplification containing only the region correspondent to mature toxin (Domains 1, II and III - 2001 bp); **lane 2**: DNAse I digestion product resulting to fragments of 50 bp; **lane 3**: Reassembled PCR product using as template fragments containing 50 bp (obtained jointly and gel purified) and no primers added to reaction; **lane 4**: PCR amplification containing pool of variants (around 2000 bp) to reassembled genes.

To generate variants by applying the DNA shuffling procedure, the *cry8Ka1 *nucleotide sequence (2001 bp) was PCR-amplified from the original vector and fragmented using DNAse I. The resulting fragments in the 30-50 bp range were jointly purified and recombined to form reassembled genes using two consecutive PCR progressive programs. Using agarose gel electrophoresis, the DNA shuffling product was visualised as a single band of around 2000 bp that represented a population of *cry8Ka1 *variant genes (Figure 1).

Both the DNA that contained the population of gene variants (DNA shuffling products) and also the PCR-amplified *cry8Ka1 *gene were excised from gel, purified and then cloned into the pCOMB3X phagemid, resulting in pCOMB*cry8Ka1 *and pCOMB*cry8Ka1*var constructs. The pCOMB*cry8Ka1*var construct was used to generate a combinatorial phage-display library of *cry8Ka1 *variants containing 1.0 × 10^5 ^cfu/mL.

### 3.2 Screening of cry8Ka1 combinatorial library towards BBMVs A. grandis

**Figure 2:**
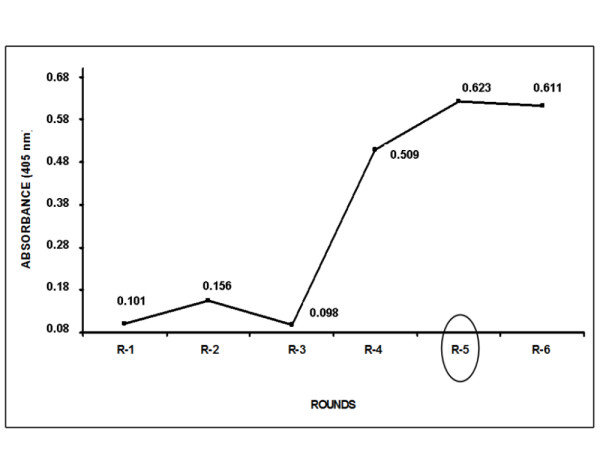
**Biopanning screening of the *cry8ka1 *Phage display combinatorial library for interactions of Cry8Ka1variants with Brush Border Midgut Vesicles (BBMVs) from cotton boll weevil larvae (*A. grandis*)**. The absorbance indicated in the graph shows the phage-infected, which was related to the quantity of bacterial colonies obtained from each round of biopanning. Based in the data, the fifth round of biopanning was chosen as cycle of the enrichment of recombinant phages displaying Cry8Ka1 variants specifically bound to *A. grandis *BBMVs. The points of the curve indicate the absorbance at 405 nm (reading of phage) in each round.

To select the clones expressing Cry8ka1 variants that bind specifically to the *A. grandis *midgut, the phage-display combinatorial library was used for biopanning. Five selection rounds were performed. The fifth round was chosen because it yielded phage showing the highest binding specificity to *A. grandis *BBMVs (Figure 2). Two hundred randomly chosen clones from the fifth round exhibited amplicons corresponding to the original gene size of around 2000 bp (data not shown). The expression of the variant proteins was confirmed by dot blot detection of the haemagglutinin (HA) epitope fused to the variant proteins. A reaction signal was observed in the dot blot for most of the analysed clones (data not shown). Thirty clones that showed an expression signal in the dot blot analyses were randomly selected for further analysis of activity against *A. grandis*.

### 3.3. Evaluation of toxicity of the Cry variants in bioassays

**Figure 3:**
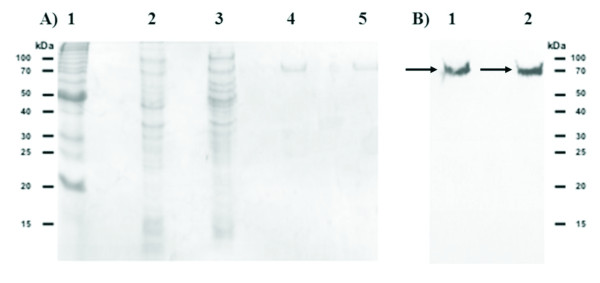
**SDS-PAGE electrophoresis and Western blot analysis of Cry8Ka1 and Cry8Ka5 expressed in *E. coli *cells**. The original and variant recombinant Cry8K toxins were fractionated by SDS-PAGE, blotted onto a nitrocellulose membrane and probed with monoclonal Anti-His^® ^antibody **A) Lane 1**, BenchMark™ Protein Ladder (Invitrogen); **lane 2**, proteins from non-induced cells (supernatant); **lane 3**, proteins expressed after induction with 0.5 mM IPTG-(supernatant); **lane 4 and 5**, Cry8Ka5 and Cry8Ka1 recombinant toxins, respectively, purified by using affinity chromatography as described in material and methods. **B) **Western immunoblot analysis. **Lane 1 and 2 **the arrows indicate Cry8Ka5 and Cry8Ka1 recombinant toxins, both with approximate molecular weights of 70 kDa.

To assess the LC_50_, neonate *A. grandis *larvae were incubated with purified Cry8Ka1 and Cry8Ka5 toxins expressed by the *E. coli *BL21 (DE3) strain (Figure 3).

**Figure 4:**
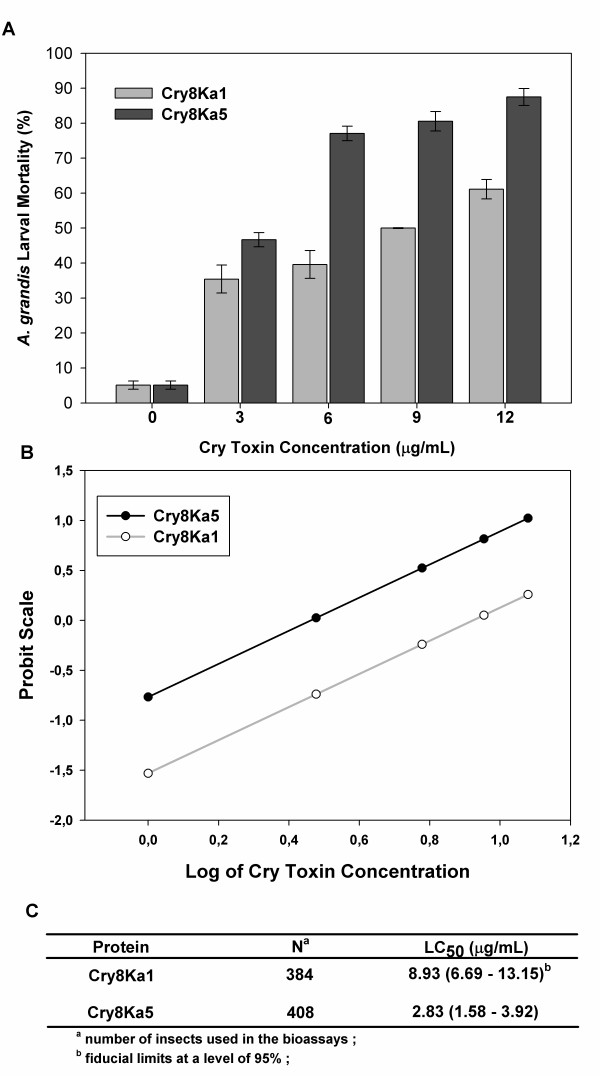
**Toxicity of Cry8Ka1 (Wild-type) and Cry8Ka5 (variant) proteins against *A. grandis***. **A**: Percentage mortality of *A. grandis *larvae at 7 days after exposure to 0, 3, 6, 9 and 12 μg/mL of Cry 8Ka1 and Cry 8Ka5. **B**: Probit Scale plotted against logarithmically Toxin Dilution (base 10). **C**: LC_50 _and fiducial limits.

Bioassays were conducted to evaluate the activity of Cry8Ka1 variants compared to the wild-type Cry8Ka1 toxin when incubated with neonate *A. grandis *larvae. The larvae mortality rate (Table 1) indicated no significant difference between treatment with a diet supplemented with Cry8Ka1 protein, a diet supplemented with M13 helper phage or a diet with no protein supplementation (negative control) at the concentration tested. On the other hand, two (Cry8Ka3 and Cry8Ka5) out the thirty Cry8Ka1 variants tested had a statistically significant effect on *A. grandis *compared to controls. The analysis of variance was significant for the mortality rates of these two variants. Based on these results, the Cry8Ka5 toxin was selected for additional characterisation (Figure 4A). The LC_50 _for the recombinant Cry8Ka5 protein was significantly lower (2.83 μg/mL) than that of Cry8Ka1 (8.93 μg/mL) (Figure 4B and 4C), indicating improved toxicity (three times higher) for the novel toxin (Cry8Ka5) selected from the combinatorial library of Cry8Ka1 variants (Figure 4C).

**Table 1:** Bioassay showing larvicidal activity including Cry8Ka1 toxin, the others toxins encoded by the seven selected gene variants and VCSM13 Phage (as negative control) using *A. grandis *larvae.

Treatment	N	Lethality Mean (%) ± SD	SEM
VCSM13 Phage (Control)	6	20.55 ± 2.50 b^1^	1.02
Cry8Ka1 (Wild-type)	5	29.33 ± 5.47 a,b	2.44
Cry8Ka2	5	36.66 ± 5.77 a,b	2.58
Cry8Ka3	5	49.33 ± 6.30 a	2.81
Cry8Ka4	6	33.88 ± 11.95 a,b	4.88
Cry8Ka5	4	50.00 ± 11.54 a	5.77
Cry8Ka6	6	32.77 ± 15.22 a,b	6.21
Cry8Ka7	6	44.44 ± 19.16 a,b	7.82
Cry8Ka8	5	45.99 ± 17.50 a,b	7.82

10 μg/ml of expressed phage were used in every treatment

**N**, number of bioassays (12 larvae/replicate); **SD**, standard deviation; **SEM**, Standard Error Mean.

^1 ^Results indicated with the same letter have no statistical difference between treatments (p < 0,05).

### 3.4 Structural analysis of Cry8Ka1 variant toxins

Firstly, the presence of genetic variability in the *cry8Ka1 *combinatorial library was determined using nucleotide sequence analysis (data not shown) of 30 clones isolated from the pool of variants that was obtained at the fifth round of biopanning, which was chosen as the cycle containing the highest enrichment of specific phage.

**Figure 5:**
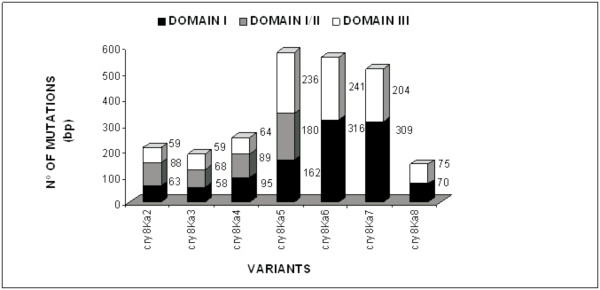
**Graphic representation indicates number of substitutions in the nucleotide sequences, distributed among the three domains for that seven selected *cry8Ka1 *variants**. The sequences of variants named *cry8Ka2, cry8Ka3, cry8Ka4, cry8Ka 5, cry8Ka6, cry8Ka7 *and *cryKa8 *were fully sequenced and aligned using *cry8Ka1*. In black color, indicate domain I, in gray color, domain II and white color, domain III. In each variant a large number of nucleotide mutations was observed. The *cry8Ka5 *variant contained 578 nucleotide substitutions in total.

**Figure 6:**
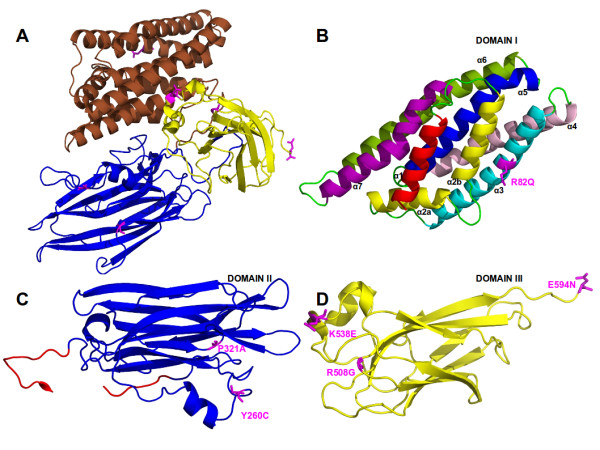
**Ribbon diagrams of the Cry8Ka1 structural model produced with PyMOL **[39]**and indicating the positions of the amino acid substitutions obtained in Cry8Ka5 toxin**. A: The whole molecule showing the three domains (Domain I in brown color, II in blue color and III in yellow color). Cry8Ka5 substituted residues are highlighted in magenta color. In B, Domain I, composed of eight α-helices, is shown. The position of the R82Q mutation is indicated on helix α3 (also indicated in Fig 7). In C, the anti-parallel β-sheets of domain II, indicating two substituted residues in Cry8Ka5 toxin: Cys and Ala in positions 260 and 321, respectively. In D, the jelly-roll β-sheets of Domain III, and the three other mutated residues: Gly at position 508, Glu at 538 and Asn at 594.

**Figure 7:**
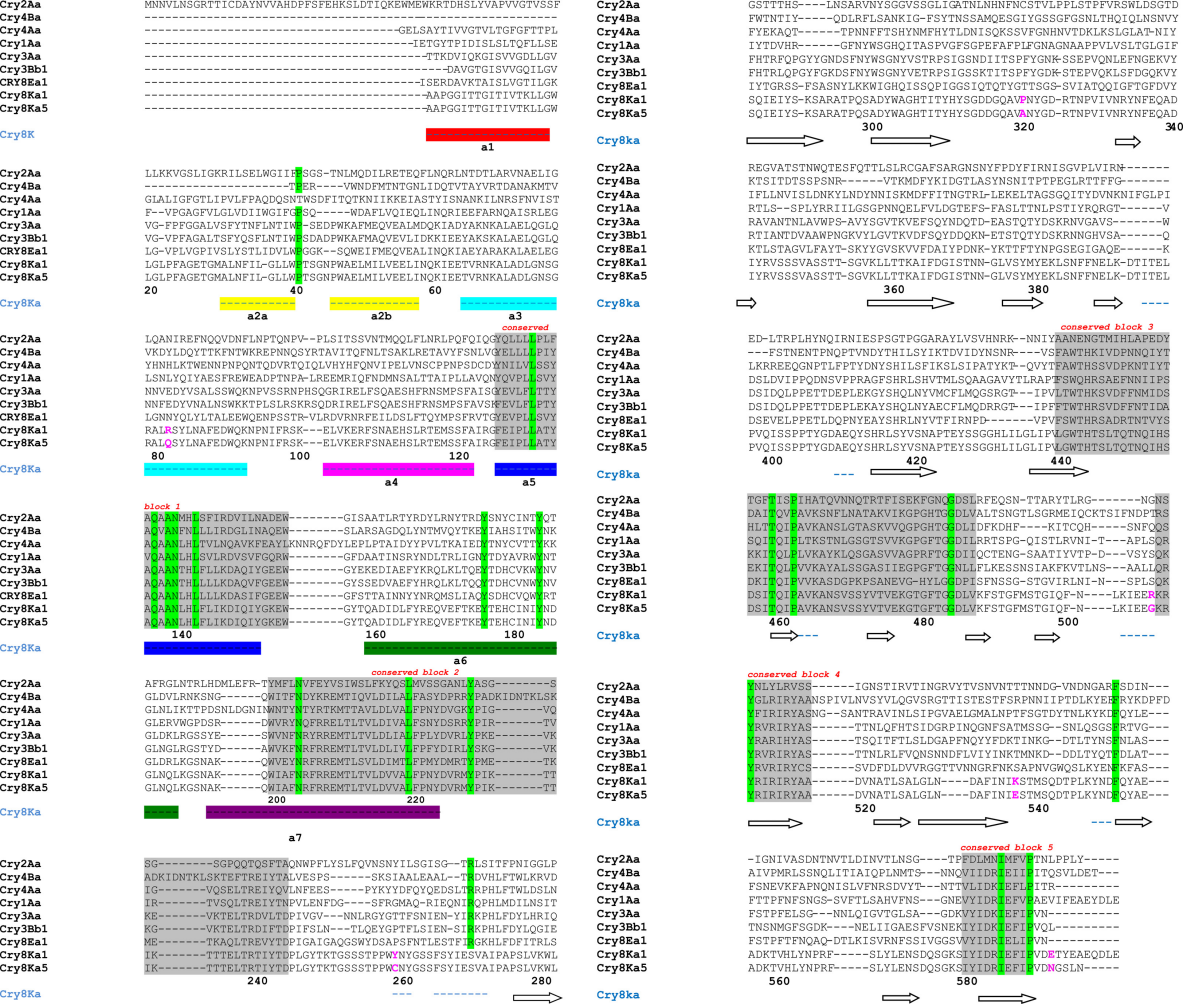
**Multiple sequence alignment of activated Cry toxins after digestion (mature toxin) including translated Cry8Ka1 and mutant Cry8Ka5 sequences**. From top to bottom the sequences are Cry2Aa (Uniprot code: P0A377), Cry4Ba (P05519), Cry4Aa (P16480), Cry1Aa (BAA04468), Cry 3Aa (P0A379), Cry3Bb1 (Q06117), Cry8Ea1 (NCBI accession: AAQ73470.1), Cry8Ka1 (FJ422558) e Cry8Ka5. The secondary structure of Cry8Ka1 is indicated in the bottom line. The Cry8Ka α-helix colors match up to the same α-helix colors presented in our homology model (Fig 6) constructed using Cry8Ea1 (PDB entry 3EB7) as template. The highly conserved boxes of Cry family are indicated by gray color and labeled in the top line. Highly conserved residues are highlighted in green. The five replaced residues in the Cry8Ka5 sequence comparing to Cry8Ka1 are highlighted in magenta. The numbers in the bottom line are those of the structural Cry8Ka1 model.

The selected *cry8Ka5 *gene (1947 bp) encoded a protein of 649 amino acid residues. Sequence comparisons among the parental *cry8Ka1 *gene (2001 bp) and the variant genes identified numerous nucleotide substitutions (Figure 5). However, most of these nucleotide substitutions were silent mutations. *In silico *amino acid sequence translation revealed just six changed residues that were located in positions distributed throughout the three typical domains of Cry toxins, using the Cry8Ka1 and Cry8Ka5 comparison as indicated (Figure 6). One substitution was observed in domain I (R82Q; within the α3 helix), two substitutions (Y260C and P321A) were observed in domain II and three substitutions (R508G, K538E and E594N) were observed in domain III. In addition to residue substitution, a deletion of 16 residues in the N-terminal part of the Cry8Ka5 protein was observed, and this deletion was responsible for the reduced size of the mutant as compared to Cry8Ka1 (not shown). Additional sequence analyses revealed that the five conserved blocks in the primary structure that were previously defined in typical Cry toxins [10,16,40] were retained in both the Cry8Ka1 parental molecule and the shuffled Cry8Ka5. According to multiple sequence alignments (Figure 7), the Cry8Ka toxins studied here had all of the highly conserved residues (highlighted in green colour) except for residue R, which was substituted to S in position 271. There seems to be no obvious functional consequence of this substitution.

In addition, molecular homology analyses performed using three-dimensional models constructed in this study showed identical folding to Cry8Ka5 such that Cry toxins were included in the group of three-domain Cry toxins. The structural alignments using Cry8Ka1 and Cry8Ka5 protein sequences indicated the three best results showing 34.7; 35.4 and 35.7% of identity with Cry8Ea1 (3EB7.pdb) [16], Cry3Aa (1DLC.pdb) [41] and Cry3Bb1 (1JI6.pdb) [42], respectively. Our proposed model for Cry8Ka toxins was obtained from structural alignments submitted to Modeller (as detailed in Materials and Methods) and is illustrated in Figure 6. The final Cry8Ka1 and Cry8Ka5 structural models were chosen based on similar and uniform distribution of amino acids in a Ramachandran plot (93% favourable residues, 6.2% residues in allowed locations; 0.8% unfavourable residues for Cry8Ka1 and 92.6% favourable residues, 6.6% residues in allowed locations; 0.8% unfavourable residues for the shuffled Cry8Ka5) and on DOPE analysis. The DOPE residue-by-residue energy profiles for the final models (not shown) suggested that the models were of high quality.

The Cry8Ka1 and Cry8Ka5 mutant models had the same backbone structure and differed only in the mutated side chains. Due to the very low sequence identity found in the N- and C-termini when compared to the template, these regions were not modelled. As shown in Figure 6, the Cry8Ka toxin model presents the three conserved domains (I, II and III) typical of Cry toxins. The positions of residues replaced in the shuffling process can be better seen in Figures 6b-d, in which the individual domain structures are illustrated. All mutations are localised at the surface and exposed to the solvent. In the Cry8Ka1 model, domain I consists of residues 1-238, domain II contains residues 239-450 and domain III includes residues 451-594.

## 4. Discussion

In this work, the *cry8Ka1 *gene was used as a substrate for DNA shuffling. This gene was isolated from the *B. thuringiensis *S811 strain [25] and codes for a 668 residue protein with the conserved three domain structural architecture typical of Cry toxins. The Cry8Ka1 toxin has been shown to be moderately toxic to *A. grandis*, and our research focused on the molecular improvement of the Cry8Ka1 toxin. The challenge was to introduce changes in the primary structure and thereby achieve improved insecticidal activity.

The combined strategy using DNA shuffling and phage display techniques resulted in a combinatorial library containing 10^5 ^variants. The efficiency of the DNA shuffling approach to improve specificity and to broaden the spectrum of insects controlled by Bt toxins was first mentioned in experiments using the *cry1Ca *gene. Screening of a *cry1Ca*-shuffled library for activity against *Spodoptera frugiperda *revealed a Bt toxin variant showing 3.8-fold higher LC_50 _when compared to the wild-type. In other experiments, Cry1Ca variants tested against *S. exigua *showed 6.7-fold higher activity than wild-type Cry1Ca. In addition, the same variants were active against both *Heliothis zea *and *S. exigua *[20].

When screening genes for naturally occurring genetic variability or for artificially generated variability as performed in this study, the major concern is the choice of an optimal selection system that guarantees efficient screening of mutants containing the desired characteristics. The technique of displaying molecules on the phage surface [43-45] has been widely applied to select antibody chains, to identify receptor ligands, to define enzyme substrates, and to select anticoagulant activities and proteinase inhibitors [46-49]. In addition, phage display systems have proven to be a useful tool for studying toxins with binding domains such as Cry toxins [24]. In the present study, we applied phage display methodology using M13 filamentous phage; we chose to couple this strategy to DNA shuffling because Cry toxins are AB-type toxins, which have been shown to be suitable targets for directed evolution [50,14]. As discussed in a previous study from our group [24], similar studies have encountered problems with the functionality of proteins displayed on M13 phage. In this study, the bioassays using Cry8ka5 recombinant protein confirmed the molecule's effectiveness and emphasised the need to establish better conditions that would overcome constraints to successfully express Cry protein variants with the M13 phage display system. The mortality found from negative control in the first screening bioassays was due the M13 phage system. The bioassays using boll weevil artificial diet as here described represent a routine in our researches aimed to investigate the potential of novel molecules against boll weevil larvae. However, it is clear that for biotechnology purpose, e.g. production of insect-resistant plants, further tests must be conducted using boll weevils in presence of the genetic modified plants containing the selected variant genes. In the present work the selected Cry8Ka5 mutant toxin with improved activity against the insect target had six residue substitutions distributed throughout the three domains and a shorter N-terminal end than the wild-type Cry8Ka1. Because the selected mutant toxin was 3.1 times more active than the parental Cry8Ka1 toxin and 81.3 times more active than the Cry1Ia12 toxin [25], we concluded that the strategy of combining DNA shuffling and phage display was able to effectively select novel toxins that are more highly active against the cotton boll weevil.

We modelled the three-dimensional structures of the Cry8Ka1 toxin and the Cry8Ka5 mutant toxin by using the three-dimensional crystal structure of activated Cry8Ea1 (3EB7.pdb) toxin [16] as templates for homology modelling. Cry8 and Cry3-type toxins are active against a number of coleopteran pests; however, Cry8Ea1 showed specific toxicity against *Holotrichia parallela *(Scarabaeidae) [16]. Although Cry coleopteran toxins have not been as extensively studied as Cry1A, it has been shown that, as for Cry1A lepidopteran-specific toxins, the pore formation activity of Cry3 toxins depends on the formation of an oligomeric pre-pore structure after the interaction of protoxins with BBMV midgut cells from the Colorado potato beetle [51]. One previously reported theory is that the helix bundle in domain I is directly responsible for membrane penetration and pore formation after Cry toxins bind the specific receptors on the surface of the insect midgut [16]. In support of this theory, our Cry8Ka toxin models confirmed the presence of a seven-helix bundle, as has been generally described, and we also visualised the helix α2 separated in two helices (Figure 7), which is conserved among Cry toxin structures [16]. According to previous authors, the α2 helix break could be related to the mobility of the lid on the helix bundle during correct receptor recognition. At that moment, the lid comprising helix α2b and loop α2b- α3 must be removed from the top of the helix bundle to release the helical hairpin α4-α5. Based on mutagenesis studies, the same authors discussed the significance of the conserved Pro-41 residue (residue number correspondent to primary structure of the Cry8Ka toxins - Figure 7) in maintaining the stability of Cry8Ea1. According to a more recent model proposed for the initiation of Cry toxin domain disassembly, Pro-41 could be the key in facilitating the lid-opening starting from a stable conformation [16]. Based on these structural analyses, we suggest that the DNA shuffling strategy applied here was able to generate mutations without causing conformational changes that could affect the stability of a new toxin. Regarding the involvement of the domains II and III in the Cry toxin mechanism, a combination of site-directed mutagenesis and membrane binding studies [52] showed that the exposed loops of domain II on Cry3A are involved in receptor binding and that mutations can affect binding, resulting in increased or decreased toxicity. Loop 1 and loop 3 in domain II of Cry3A are directly involved in receptor binding. In addition, the same authors suggested that the loop III region might play a role in irreversible binding or membrane insertion. One of the mutations created by the cry8Ka1 gene shuffling procedure is localised in loop 3 of domain II in Cry8Ka5. However, neither of the two mutations in domain II of Cry8Ka5 is located in these loops.

Our results indicated that most structural features of the highly conserved residues are present in the Cry8Ka modelled structures. Given that our experiments to determine the specific receptors for Cry8Ka1 toxins are currently in development, the use of the molecular modelling results to explain how differences in toxicity could be caused by the specific residues changes is somewhat speculative. A cDNA library constructed using intestinal material from cotton boll weevil larvae will be used to screen receptor proteins. We hope that studies involving receptor-toxin interactions will allow us to elucidate the binding differences and increased toxicity related to the replaced residues in the three domains of the Cry8Ka5 mutant.

## 5. Conclusion

In conclusion, we have demonstrated that the strategy of combining DNA shuffling and phage display was able to effectively select novel toxins that are more highly active against the cotton boll weevil. With its improved toxicity against the cotton boll weevil, a major cotton insect-pest, the *cry8Ka5 g*ene has the potential to be used in plant transformation experiments to produce genetically modified cotton plants that are *A. grandis*-resistant. Our modelling studies show conservation of core residues in the Cry8Ka5 toxin structure, suggesting that the mutations altering activity should not affect molecular stability.

## Authors' contributions

GRO have carried out all experiments involving in vitro directed molecular evolution studies, and also carried out experiments aiming overexpression of recombinant proteins and its application into bioassays; MCMS carried out design and biopanning experiments, molecular modeling studies and involved in drafting the manuscript; WAL participated of constructions of structural models and drafted the manuscript; EYTN contributed to molecular characterization of the selected molecules; AAPF participated of sequence analysis, protein modeling, and have been involved in drafting the manuscript; MAB carried out the immunoassays; DSLS contributed to analysis and interpretation of data; JEGJ carried out BBMVs proteins preparation and participated to Phage display experiments; JDASJ participated in the design of the study, sequence analysis, vector construction for bacterial expression, drafting the manuscript and performed the statistical analysis DJR has been participated in the molecular structural studies and involved in revising of the manuscript critically; HBR participated of DNA shuffling experiments and bioassays; CRS coordinated studies and obtain recombinant protein expression; MFGS coordination of the research group carried out all design experiments and data analysis. All authors read and approved the final manuscript.
